# Neuropathogenesis of SARS-CoV-2 infection

**DOI:** 10.7554/eLife.59136

**Published:** 2020-07-30

**Authors:** Shumayila Khan, James Gomes

**Affiliations:** Kusuma School of Biological Sciences, Indian Institute of Technology DelhiNew DelhiIndia; Icahn School of Medicine at Mount SinaiUnited States; University of ZurichSwitzerland

**Keywords:** SARS-CoV-2, COVID-19, neurotropic behaviour, neurological disorder, brain, stroke, Human

## Abstract

The COVID-19 pandemic caused by the SARS-CoV-2 has recently emerged as a serious jolt to human life and economy. Initial knowledge established pulmonary complications as the chief symptom, however, the neurological aspect of the disease is also becoming increasingly evident. Emerging reports of encephalopathies and similar ailments with the detection of the virus in the CSF has elicited an urgent need for investigating the possibility of neuroinvasiveness of the virus, which cannot be ruled out given the expression of low levels of ACE2 receptors in the brain. Sensory impairments of the olfactory and gustatory systems have also been reported in a large proportion of the cases, indicating the involvement of the peripheral nervous system. Hence, the possibility of neurological damage caused by the virus demands immediate attention and investigation of the mechanisms involved, so as to customize the treatment of patients presenting with neurological complications.

## Introduction

The severe acute respiratory syndrome coronavirus 2 (SARS-CoV-2), has caused the outbreak of a global pandemic with the first breakout reported in the Hubei province of Wuhan. Although the clinical manifestation of the COVID-19 disease caused by the virus predominantly involves the respiratory system, recent reports suggest that the pathology of the virus may extend beyond it to involve other organ systems such as the brain and nervous system as well. Therefore, understanding these impairments caused by the SARS-CoV-2 is of utmost importance, so that the treatment of the patients with atypical symptoms can be tailored accordingly. Since the penetration of the virus into the brain can result in long-term debilitating conditions, the opportunity for systematic recording of clinical data of such patients to support research efforts should not be missed.

## SARS-CoV-2 and ACE2 receptors in the brain

The Angiotensin-converting enzyme 2 (ACE2) has been identified as the functional receptor for the SARS-CoV-2, which uses its Spike (S) protein C-terminal domain (CTD) to attach to the ACE2 receptor (Wang et al., 2020a). This study showed that the SARS-CoV-2-CTD has substantially more van der Waal contacts than the receptor binding domain of SARS-CoV, and hence exhibits higher binding affinity for ACE2. The distribution of ACE2 in various organs in humans, is mostly known from a study that used RNA obtained from 72 tissue samples of 3 donors. It was observed that although ACE2 was not as ubiquitous as ACE, low levels of ACE2 mRNA were detected in the human brain using quantitative real-time RT-PCR (Harmer et al., 2002). A later study (Hamming et al., 2004) investigated the distribution of ACE2 protein in various human organs. Tissue from 15 organs obtained from patient biopsies, unused donor organs and autopsy brain tissue was probed through immunohistochemistry. It reported that the ACE2 protein was expressed abundantly in lung and small intestine tissues, and also in venous endothelial and arterial smooth muscles of all organs, including the brain. Taken together, the presence of ACE2 in all organs and higher binding affinity of SARS-CoV-2-CTD for ACE2 are worrisome, particularly considering the mutations being detected across demographic communities (Cao et al., 2020).

## Encephalitis and encephalopathies

A preliminary evidence regarding the neurological complications due to the SARS-CoV-2 came from a report from Wuhan, which stated that 36.4% of 214 screened patients of COVID-19 displayed some form of neurological symptoms. Out of the 214 patients screened, 88 had severe infection and showed higher incidence of the neurological symptoms such as acute cerebrovascular diseases [5.7%] impaired consciousness [14.8%] and skeletal muscle injury [19.3%] (Mao et al., 2020). One study from Japan which described the first case of COVID-19-associated encephalitis where the patient was admitted for convulsions accompanied by unconsciousness reported that although the patient tested negative for SARS-CoV-2 in a nasopharyngeal swab, the viral RNA was surprisingly detected in the CSF, and the patient MRI exhibited abnormalities of the medial temporal lobe and hippocampus (Moriguchi et al., 2020). A similar report of encephalopathy emerged from China where the viral RNA was also detected in the CSF (Beijing Ditan Hospital, 2020). It has been reported that a patient from USA, later diagnosed with COVID-19, had exhibited altered mental status. Clinical investigations confirmed acute necrotizing haemorrhagic encephalopathy (Poyiadji et al., 2020). Since the CSF was limited, SARS-CoV-2 analysis could not be performed but the brain MRI images showed lesions in the sub-insular regions, and the thalamus and medial temporal lobes. An elderly patient from Iran, who tested positive to COVID-19, had suffered a severe intracerebral haemorrhage and was presented unconscious. (Sharifi-Razavi et al., 2020). It appears that a correlation exists between COVID-19 patients and the concomitant encephalopathy and cerebropathy observed without any obvious cause other than the viral infection.

There is also a report of a COVID-19 patient with Parkinson’s Disease who presented with focal dysfunction of the left temporal lobe and altered mental status (Filatov et al., 2020). This raises the question whether pre-existing disorders of the nervous system may play a role in exacerbating the neurological aspect of the disease and may be a risk factor for increased pulmonary complications. On the other hand, it will also be interesting to know whether SARS-CoV-2 leads to an increase in the incidence of neurodegenerative disorders in the affected individuals in the future (De Felice, 2019). Nevertheless, hypoxic injury to the brain caused by respiratory failure in COVID-19 patients is of serious concern. In this context, hypoxic encephalopathy was reported in 20% of 113 deceased COVID-19 patients in China (Chen et al., 2020). Recently, a detailed study carried out by a multidisciplinary group from the National Hospital in London, examined data from SARS-CoV-2 positive patients who exhibited neurological problems. The clinical, radiological, laboratory and neuropathological findings of 43 qualifying patients were studied retrospectively by the team. The categories of neurological complications included encephalopathies (10/43), inflammatory CNS syndromes (12/43), ischaemic stroke (8/43), peripherical neurological disorders (8/43) and miscellaneous central disorders (5/43) (Paterson et al., 2020). This study makes available patients’ vignettes in these categories that document the progression of the neurological problem, clinical intervention and patient response. Thus, reports such as these, suggest that the SARS-CoV-2 may be capable of inflicting injury to the brain in different ways. In Table 1, a list of neurological symptoms observed has been presented.

**Table 1.** Neurological manifestations of SARS-CoV-2 infection.

Neurological manifestations	Observations	Supportive neuro-diagnostic measures	Treatment	References
Stroke	Ischaemic stroke, deep vein thrombosis	MRI, ECG, EKG; blood test for coagulation factors, inflammatory markers	Antiplatelet, anticoagulant, tissue plasminogen activator, intravenous thrombolysis	Mao et al., 2020, Beyrouti et al., 2020, Zhou et al., 2020a, Li et al., 2020
Seizure	Generalized seizures, convulsions, tonic-clonic seizures, status- epilepticus	MRI, CT scan, EEG; CSF for viral presence	Anti-epileptic medication Levetiracetam, Clonazepam and Valproate	Mao et al., 2020; Duong et al., 2020; Sohal and Mansur, 2020; Bernard-Valnet, 2020
Encephalopathy	Disorientation, confusion hallucinations, altered mental status, agitation, irritability, dissociated speech, lethargy	MRI, EEG; CSF for viral presence	Treatment for related problems such as seizures	Mao et al., 2020; Poyiadji et al., 2020; Duong et al., 2020; Yin et al., 2020; Helms et al., 2020,
Encephalitis/meningitis	Focal neurological defects, fever, headache, other neuropsychotic symptoms	MRI, EEG; CRP levels for inflammation; CSF for viral presence	Antivirals such as Acyclovir, Favipiravir, antibiotics such as Ceftriaxone, Vancomycin, and steroids	Mao et al., 2020; Bernard-Valnet, 2020; Chaumont et al., 2020; Hayashi et al., 2020
Anosmia and ageusia	Reduction or loss of taste and smell, without nasal obstruction	Self diagnosis	Usually no treatment	Mao et al., 2020; Lechien, 2020; Hawthorn, 2018; Fotuhi et al., 2020; Galougahi et al., 2020; Spinato et al., 2020
Brain haemorrhage	Intracranial blood loss, haemorrhagic rim enhancement in multiple parts of the brain	MRI, CT scan; CSF for viral presence	Intravenous immunoglobulin	Mao et al., 2020, Poyiadji et al., 2020
Myalgia, partial paralysis and/or Guillain-Barré syndrome	Facial drooping, muscle weakness, diplegia/tetraplegia or hemiplegia; GBS-progressive, ascending, symmetrical flaccid limb paralysis, along with areflexia or hyporeflexia with or without cranial nerve involvement	MRI, CSF evaluation, CPK levels for muscle tissue damage, test for anti-ganglioside antibodies, nerve conduction studies for demyelinating neuropathy evaluation	Intravenous immunoglobulin, physiotherapy	Wang et al., 2020b; Toscano et al., 2020; Sedaghat and Karimi, 2020; Oxley et al., 2020; Zhou et al., 2020a; Zhao et al., 2020; Camdessanche et al., 2020

## Cerebrovascular disease and stroke

COVID-19-related stroke is becoming an increasing cause of concern among clinicians, particularly for patients having comorbidities like obesity, diabetes, hypertension, and heart disease. In Tongji Hospital, Wuhan, 449 severe COVID-19 patients were recruited from among 1786 consecutive patients to study coagulopathy-associated stroke. The study used sepsis-induced coagulopathy (SIC) along with elevated D-dimer levels to show that administering heparin to severe COVID-19 patients resulted in better prognosis (Tang et al., 2020). In the study by Mao et al., 2020, acute cerebrovascular diseases was reported in 5.7% patients with severe infection. Another study with 219 COVID-19 patients found that 10 (4.6%) suffered from ischaemic stroke and 1 (0.5%) had developed intracerebral haemorrhage. The stroke patients also showed higher levels of inflammatory markers like C-Reactive Protein and D-dimer (Li et al., 2020). Elevated D-dimer levels (>1000 μL) were also reported in the study at National Hospital, London where six COVID-19 patients who suffered from ischaemic stroke presented with large vessel occlusion (Beyrouti et al., 2020). These patients also exhibited higher levels of ferritin and lactate dehydrogenase usually reported in severe cases. Although a causal relationship between COVID-19 and ischaemic stroke could not be confirmed because of competing conditions, the infection was linked to the prothrombic state conducive to stroke. Another retrospective study examined the radiological evidence of 4 patients and attributed the cerebrovascular accident to likely hypercoagulability; elevated D-dimer was also reported in these cases (Avula et al., 2020). Records of 3556 COVID-19 patients hospitalized in the New York metropolitan area were inducted in a retrospective study. Among these, the data from 32 patients having proven ischaemic stroke was analysed. This detailed study included categorization of study variables, ASCOD classification of stroke and statistical analyses. A majority of the strokes reported in this study were cryptogenic and correlated to acquired hypercoagulability (Yaghi et al., 2020). There have been numerous other reports of COVID-19-related strokes as well (Zhou et al., 2020a). However, it is still a matter of debate if these strokes are a direct cause of viral infection to the cerebrovascular system.

## Anosmia and ageusia

Although the major proportion of symptoms reported earlier were respiratory, recent findings from different sources suggest sensory impairments, specifically anosmia and ageusia, as prevalent symptoms of COVID-19. A recent study reported that smell and taste loss were experienced in 68% and 71% of 59 COVID‐19 positive patients, respectively. It was observed that these impairments were ten times more likely in ambulatory COVID-19 positive patients. Fortunately, spontaneous improvement in these symptoms was observed in a majority of the cases which correlated well with the clinical recovery (Yan et al., 2020). One study from Italy reported these chemosensitive symptoms in 73.6% of 72 patients treated at the University Hospital of Sassari (Vaira et al., 2020). Another independent multicenter European study involving 417 COVID-19 patients, with mild-to-moderate symptoms and mean age 36.9 ± 11.4 years, reported olfactory and gustatory impairments in 85.6% and 88.0% of patients, respectively. Interestingly, this study found that females were more likely to develop COVID-19 associated anosmia and ageusia as compared to males, although the reason for this is unclear (Lechien, 2020). Also, these dysfunctions were more prevalent among European patients. These findings have been supported by epidemiologists and medical professionals from locations such as Paris, Geneva (Gautier and Ravussin, 2020) and Wuhan (Mao et al., 2020). Clinical observations across countries have put on record that anosmia and ageusia are early symptoms of COVID-19 infection (Table 1). It may be well argued that viruses in general, and SARS-COV-2 in particular, can infect peripheral neurons and subsequently gain access to the CNS. Thus, ambulatory patients presenting with chemosensory impairments during this pandemic, with otherwise mild-to-moderate symptoms, must be tested for SARS-CoV-2 infection.

## Possible mechanisms for neurological damage

**Figure 1.**
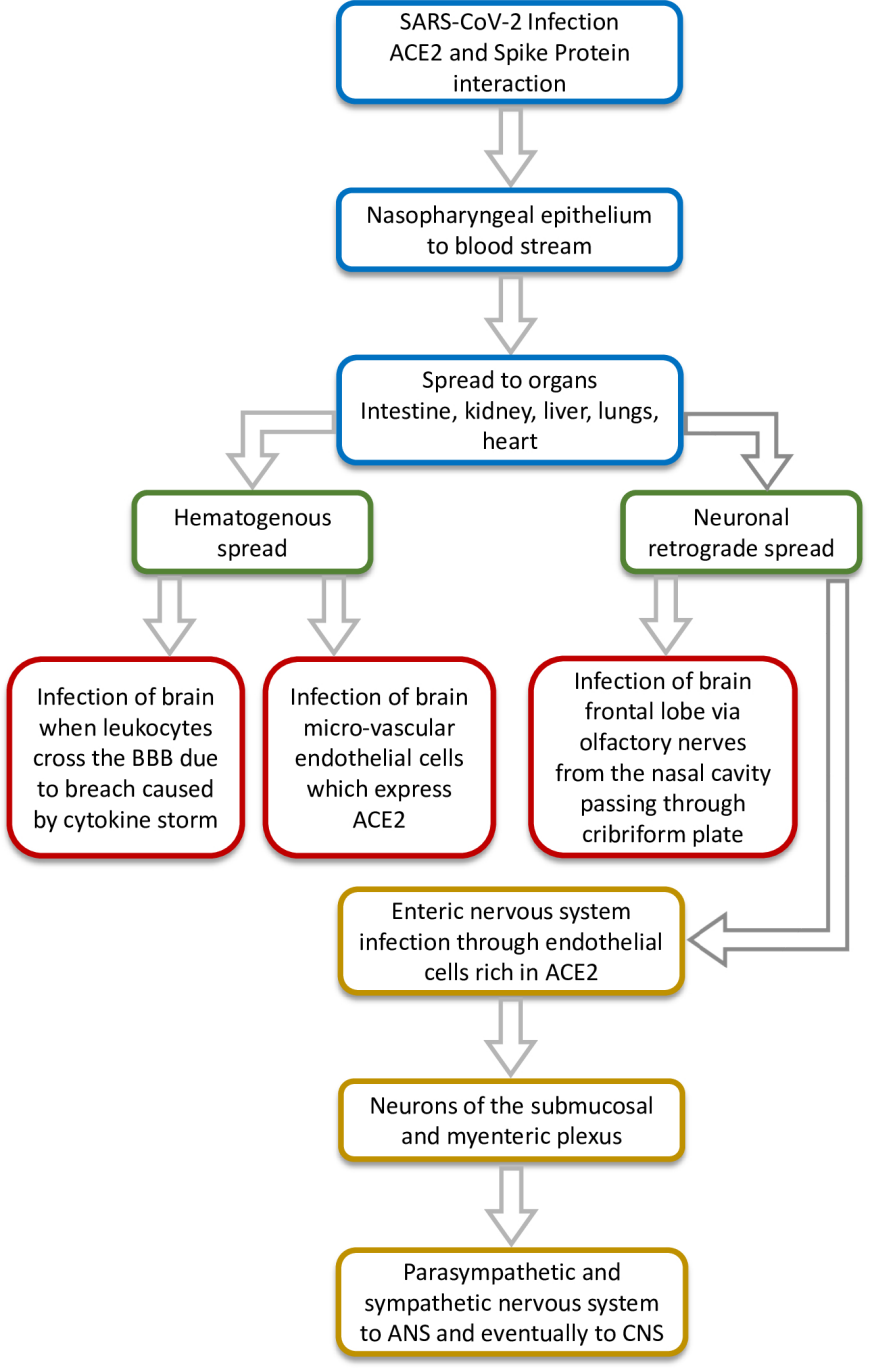
Schematic diagram showing the possible routes of neuroinvasiveness of SARS-CoV-2.

Previously, the organ distribution of SARS-CoV in 22 different tissue-types collected from four SARS and four non-SARS control autopsies were analysed. The four fatal SARS patient samples had shown the presence of SARS-CoV in the cerebrum, establishing neurotropic behaviour of the virus. Since both SARS-CoV and SARS-CoV-2 mediate host cell entry through ACE2 receptor, the presence of SARS-CoV-2 in the CSF of some patients is troubling and suggests the possibility of neuroinvasive potential of the virus; however, extensive studies are needed to confirm this. Viremia has been reported in the case of SARS-CoV-2 and it is possible that the virus may be able to spread hematogenously and breach the blood-brain barrier due to the cytokine storm reported as a pathology (Hirano and Murakami, 2020). The ACE2 receptors present on the capillary endothelium may facilitate this process. Although the exact route of entry into the CNS still remains unclear, Hoffmann et al., 2020 have demonstrated that besides the ACE2 receptor, SARS-CoV-2 uses TMPRSS2 as well as endosomal cysteine proteases to assist S-protein binding. The neurotropic transgression most likely occurs via olfactory aspiration. For instance, the brain was a major target organ of SARS-CoV in mice transgenic for the human ACE2 receptor (Tseng et al., 2007), and the virus infected the brain via the olfactory bulb, spreading quickly to the other parts by extensive neuronal infection (Netland et al., 2008). This is understandable, given the anatomy of the nasal cavity and its proximity to the forebrain, which may act as a route of entry for the virus (Figure 1). If the virus breaches the blood-brain-barrier and disseminates into the brain tissue, its spread will probably be mediated by the recruitment of pathways with specific neurotransmitters across the cerebrum. The virus can also spread via neuronal retrograde mechanism where it invades the peripheral neurons but ultimately gains entry to the brain and CNS by spreading through the connected network of neuronal transport machinery (Zhou et al., 2020b). Recently, the possibility of neuroinvasion via the enteric pathway has also come to light which suggests that the virus may prime the enteric nervous system or reach the CNS via intestinal vagal afferents (Esposito et al., 2020). The possible routes of neuroinvasion have been depicted in Figure 1. Whatever maybe the route of neuroinvasion, it is clear that the effects can be debilitating in some severe cases. Another consideration is that since neurodegenerative disorders sometimes take more than a decade to manifest, the long-term pathophysiological outcomes of the SARS-CoV-2 neurotropism should be considered as a matter of serious concern by researchers in this field.

## Prospects of future research

The ever-expanding list of cases which report nervous system complications in COVID-19 patients strongly suggests the possibility of the virus invading the brain and the nervous system. However, the fact that there is no clear evidence of how the virus mediates neuroinvasion, leaves the door open to probe this question with the help of animal models. Earlier, some studies on the transgenic rodent models, which expressed human ACE2 receptors, had unravelled the mechanisms of SARS-CoV neurotropism (Tseng et al., 2007; Netland et al., 2008). Such studies will serve as a basis to investigate if SARS-CoV-2 also employs the same pathways and strategies as SARS-CoV to infect the brain. Whether or not the virus can use the low levels of ACE2 receptors in the brain to infect the neurons needs to be investigated. Also, since the virus has been detected in the CSF of many COVID-19 patients, its ability to thrive and replicate in the CSF needs to be examined thoroughly. The possible effects of the virus on different parts of the brain, spinal cord and peripheral nervous system should also be evaluated so that the pathophysiology behind the neurological symptoms observed in patients can be understood in greater detail. This understanding will show the way forward for the development of targeted treatment regimens according to the symptoms manifested. As the clinicians are becoming increasingly aware of the neurological complications due to SARS-CoV-2, the diagnostic procedures should not be solely focused around the pulmonary manifestations, but include neurodiagnostic tests in cases where neurological symptoms are observed. The data collected can then be analysed to identify the precise pathological mechanisms employed by the virus. The RNA of the virus particles isolated from the CSF or post-mortem brain tissue samples should also be sequenced so that phylogenetic analysis can shed light on the possibility of any tissue-specific compartmentalization of the virus. Furthermore, since the manifestation of Guillain-Barré syndrome in some COVID-19 patients also hints at possible immune system disruptions caused by the virus, the effect of the virus on the neuroimmune system should be thoroughly investigated.

## Conclusion

The COVID-19 pandemic has become a challenge for the healthcare workers and researchers worldwide, as not much is known about the pathophysiology of the virus and the strategies associated with its infection. The pulmonary aspect of the disease is largely known and accepted but due to the recently emerged possibility of its neuro-invasive nature, several unanswered questions have surfaced. Autopsies of deceased patients and thorough examination of the brain and spinal cord tissues may shed some light on this topic. Moreover, probing the CSF for the presence of viral RNA, whenever possible or suspected may provide a better picture of its mechanisms of invasion. The preliminary reports which hint towards the involvement of the CNS imply an urgent need for more studies, and a systematic collection and preservation of CSF samples along with associated clinical data, at least in patients displaying extrapulmonary or neurological symptoms, to examine the neuronal aspect of COVID-19. As the neurological aspect of the viral infection remains very poorly understood, there is an immediate need to carry out experimental studies involving animal models of the disease to investigate the possible mechanisms by which the virus can gain entry into the brain, as well as cause deleterious effects on the nervous system.
